# Population Priorities for Successful Aging: A Randomized Vignette Experiment

**DOI:** 10.1093/geronb/gby060

**Published:** 2018-06-06

**Authors:** Elise Whitley, Michaela Benzeval, Frank Popham

**Affiliations:** 1 MRC/CSO Social and Public Health Sciences Unit, University of Glasgow, UK; 2 Institute for Social and Economic Research, University of Essex, UK

**Keywords:** Attitudes, Cognition, Health, Interpersonal relations, Successful aging

## Abstract

**Objectives:**

Aging populations have led to increasing interest in “successful aging” but there is no consensus as to what this entails. We aimed to understand the relative importance to the general population of six commonly-used successful aging dimensions (disease, disability, physical functioning, cognitive functioning, interpersonal engagement, and productive engagement).

**Method:**

Two thousand and ten British men and women were shown vignettes describing an older person with randomly determined favorable/unfavorable outcomes for each dimension and asked to score (0–10) how successfully the person was aging.

**Results:**

Vignettes with favorable successful aging dimensions were given higher mean scores than those with unfavorable dimensions. The dimensions given greatest importance were cognitive function (difference [95% confidence interval {CI}] in mean scores: 1.20 [1.11, 1.30]) and disability (1.18 [1.08, 1.27]), while disease (0.73 [0.64, 0.82]) and productive engagement (0.58 [0.49, 0.66]) were given the least importance. Older respondents gave increasingly greater relative importance to physical function, cognitive function, and productive engagement.

**Discussion:**

Successful aging definitions that focus on disease do not reflect the views of the population in general and older people in particular. Practitioners and policy makers should be aware of older people’s priorities for aging and understand how these differ from their own.

Industrialized populations are aging, (Christensen et al., 2009) prompting debate about whether growing proportions of older individuals require increasing investment in health and long-term care.(Bloom et al., 2015) Early research and policy often concentrated on more unfavorable aspects of aging, particularly at the population level, resulting in anxiety and negativity about its potential impact on society (Baltes & Carstensen, 1996). However, more recent evidence suggests that, compared with their peers in previous cohorts, older people today have better physical and cognitive functioning (Christensen et al., 2013; Vaupel, 2010) and are more likely to be in paid employment (Spijker & MacInnes, 2013) or volunteering (Morrow-Howell, 2010), resulting in a growing interest in the notion of “successful aging” (Araújo, Ribeiro, Teixeira, & Paúl, 2016; Bowling, 2007; Katz & Calasanti, 2015; Martin et al., 2015; Martinson & Berridge, 2015; Nimrod & Ben-Shem, 2015; Stowe & Cooney, 2015). In addition, older people are often more positive about the aging process than those involved in their care, demonstrating high levels of adjustment, acceptance, and resilience (Manning, Carr, & Kail, 2016). These views are consistent with recent challenges to the current World Health Organization (WHO) definition of health as “a state of complete physical, mental and social well-being,” which recommend an alternative formulation in terms of individuals’ ability to adapt and self-manage (Huber et al., 2011). However, there is a danger that the attitudes of practitioners and policy makers involved with older people are based on out-of-date and potentially misleading information and differences in the beliefs of older people and professionals are particularly pertinent in the context of shared decision making and patient-centered care. Although the value of patients’ opinions in shaping and informing clinical practice is well recognized in principle, recent results from the MAGIC (Making Good Decisions in Collaboration) programme (Joseph-Williams et al., 2017) highlight that, in practice, some clinicians “fail to recognize that patients’ values, opinions or preferences … may differ from their own.” Moreover, the authors report that older people may be particularly reluctant to share their views. If policy and practice are to support people to age successfully, a greater understanding of the extent to which people value different aspects of aging is required.

Clinicians, researchers, and policy makers worldwide agree that “successful aging” is an important goal (Bloom et al., 2015; Commission of the European Communities, 2009; United Nations, 2002) but its meaning remains unclear. A wide array of successful aging definitions have been proposed in the literature (Lupien & Wan, 2004). Some focus on specific domains, for example, biomedical aging, covering compression of morbidity and genetic factors, while others consider cognitive or psychosocial aging, with an emphasis on subjective well-being and personality. While these models provide insights into particular aging processes and are valuable in developing the specific policies that underpin them, they can also be limited in their ability to predict or explain other aspects of aging and this has led to the development of multidimensional models that include multiple aging dimensions.(Lupien & Wan, 2004) Again, many different multidimensional models have been proposed, some focusing on successful aging as an adaptive process, such as the Selection, Optimization and Compensation (SOC) model proposed by Baltes and Baltes (Baltes & Baltes, 1990), and others focusing on successful aging as measureable state, such as the MacArthur model proposed by Rowe and Kahn (Rowe & Kahn, 1997). There are also differences in multidimensional models proposed by different groups. For example, while the majority of operational definitions of successful aging include physiological factors such as disease, disability, and physical function (Cosco et al., 2014a; Depp & Jeste, 2006), considerably fewer include dimensions known to be of value to older people, such as functioning, social engagement, well-being, independence, and acceptance (Cosco et al., 2013). This disparity is evidenced by a number of studies indicating that many older people who consider themselves to be aging successfully do not meet clinician/researcher-defined criteria (McLaughlin, Jette, & Connell, 2012; Montross et al., 2006; Strawbridge, Wallhagen, & Cohen, 2002; Young, Frick, & Phelan, 2009). In spite of decades of research, there is still no firm consensus as to what successful aging entails, with recent special issues of *Journals of Gerontology: Social Sciences* (Pruchno & Carr, 2017) and *The Gerontologist* (Pruchno, 2015) devoted to the question. In addition, the development of appropriate metrics has been identified as a research priority by WHO (World Health Organisation, 2015). However, the most widely adopted multidimensional model of successful aging was proposed by Rowe and Kahn (Rowe & Kahn, 1997) and incorporates six dimensions: (a) avoidance of disease; (b) avoidance of disability; (c) maintenance of good physical function; (d) maintenance of good cognitive function; (e) good interpersonal social engagement (contacts and transactions with others); and (f) good productive engagement (engagement in activities of value to society such as working or volunteering). Conventionally, according to this definition an individual is considered to be aging successfully if they meet all six criteria. This straightforward characterization moves beyond the biomedical to include social and productive engagement, which have been shown to be of substantial importance to older people (Bowling, 2007; Cosco, Prina, Perales, Stephan, & Brayne, 2013; Depp, Glatt, & Jeste, 2007), and positive associations have been reported between this definition of successful aging and self-reported well-being (Strawbridge et al., 2002), health, and life satisfaction (Whitley, Popham, & Benzeval, 2016) in older people. However, the extent to which it reflects perceptions of successful aging in the general population continues to be widely debated (Bowling & Iliffe, 2011; Ferri, James, & Pruchno, 2009; Martinson & Berridge, 2015; Montross et al., 2006; Phelan, Anderson, LaCroix, & Larson, 2004; Stowe & Cooney, 2015; Strawbridge et al., 2002; Young et al., 2009) and the relative importance of each dimension is unknown. Rowe and Kahn (Rowe & Kahn, 2015) have also entered this debate, acknowledging the limitations of their model but supporting the notion that “its extensive use in scientific enquiry warrants modification over disposal.” In their discussion, they propose new priorities for research, including the need to take a lifecourse perspective to aging, to focus more on the potential benefits of an aging society, and to consider successful aging not only at the level of the individual but also at the level of society.

In order to promote successful aging at the societal level, it is vital to understand what the general population consider to be successful aging. Rather than propose another new successful aging model for additional debate, we aim instead to understand population attitudes toward the most commonly employed existing model with a view to identifying potential modifications that might make it more relevant to the general population. Existing work aimed at understanding how the general population regard successful aging has been primarily qualitative, considering responses to open-ended questions such as “How would you define successful aging?” (Bowling, 2007; Cosco et al., 2013), or asking participants to rank lists of researcher-defined dimensions (Cosco et al., 2014b; Depp et al., 2007). While results from these studies are useful, these approaches are not sufficiently systematic or robust to make inferences about the general population. An alternative, well-recognized approach is to use standardized vignettes (descriptions of a fictitious third party) in which factors used in the description are randomized to assess their relative impact on individuals’ responses, independent of their own characteristics (Auspurg & Hinz, 2015). In our experiment, participants in a large U.K. population sample were asked to rate the successful aging of a (hypothetical) third party. This approach has not, to our knowledge, been used previously in this context and provides a unique, unconfounded, empirical assessment of the relative importance of different dimensions of successful aging to the general population. In addition, very few existing studies consider how views of successful aging differ between men and women or younger and older people (Charbonneau-Lyons, Mosher-Ashley, & Stanford-Pollock, 2002; Collings, 2001; Cosco et al., 2015; Jopp et al., 2015) and there is therefore very limited information about wider societal attitudes to aging and older people, which is likely to have substantial influence on policy discussions. There is also evidence that individuals’ attitudes to aging change as they grow older (Phelan et al., 2004; Tate, Swift, & Bayomi, 2013), although the nature of these age-related changes is not well understood. Our study population includes respondents aged 16 years and over, allowing exploration of perceptions of successful aging throughout the lifecourse and according to respondent characteristics. Existing evidence in this regard is very limited. However, evidence from the medical sociological literature on lay concepts of health (Blaxter, 1990) suggests that, for example, older people might be more likely to prioritize functioning while younger individuals might focus on disease and that men might focus on physical aspects of disease while women will be more concerned with social factors. Our research aims were to gain a greater understanding of societal views of successful aging by: (a) determining the relative importance placed by the general population on the six Rowe-Kahn dimensions of successful aging and (b) understanding how perceptions of aging vary according to respondent characteristics such as age and gender.

## Methods

The Understanding Society Innovation Panel (IP) (Jäckle, Gaia, Al Baghal, Burton, & Lynn, 2017) is a stratified, geographically clustered sample of postcode sectors in Great Britain (south of the Caledonian Canal) with random selection of addresses within each sampled sector. It is designed to be representative of the British population. All household members over 16 years are invited to take part annually with refreshment samples added at waves 4 and 7. Each wave carries a number of experiments based on an annual competition and the current vignette experiment was included in the 9th (IP9). At IP9 one-third of the sample was allocated to face-to-face interviewing and two-thirds to sequential mixed mode (households were first offered a web interview and, if they did not take this up, were then allocated a face-to-face interview) with mop-up interviews carried out by telephone. Respondents are given a financial incentive to thank them for taking part. Ethics review is conducted by the University of Essex Ethics Committee. Full details of the design and experiments in IP9 can be found in (Jäckle et al., 2017).

**Figure 1:**
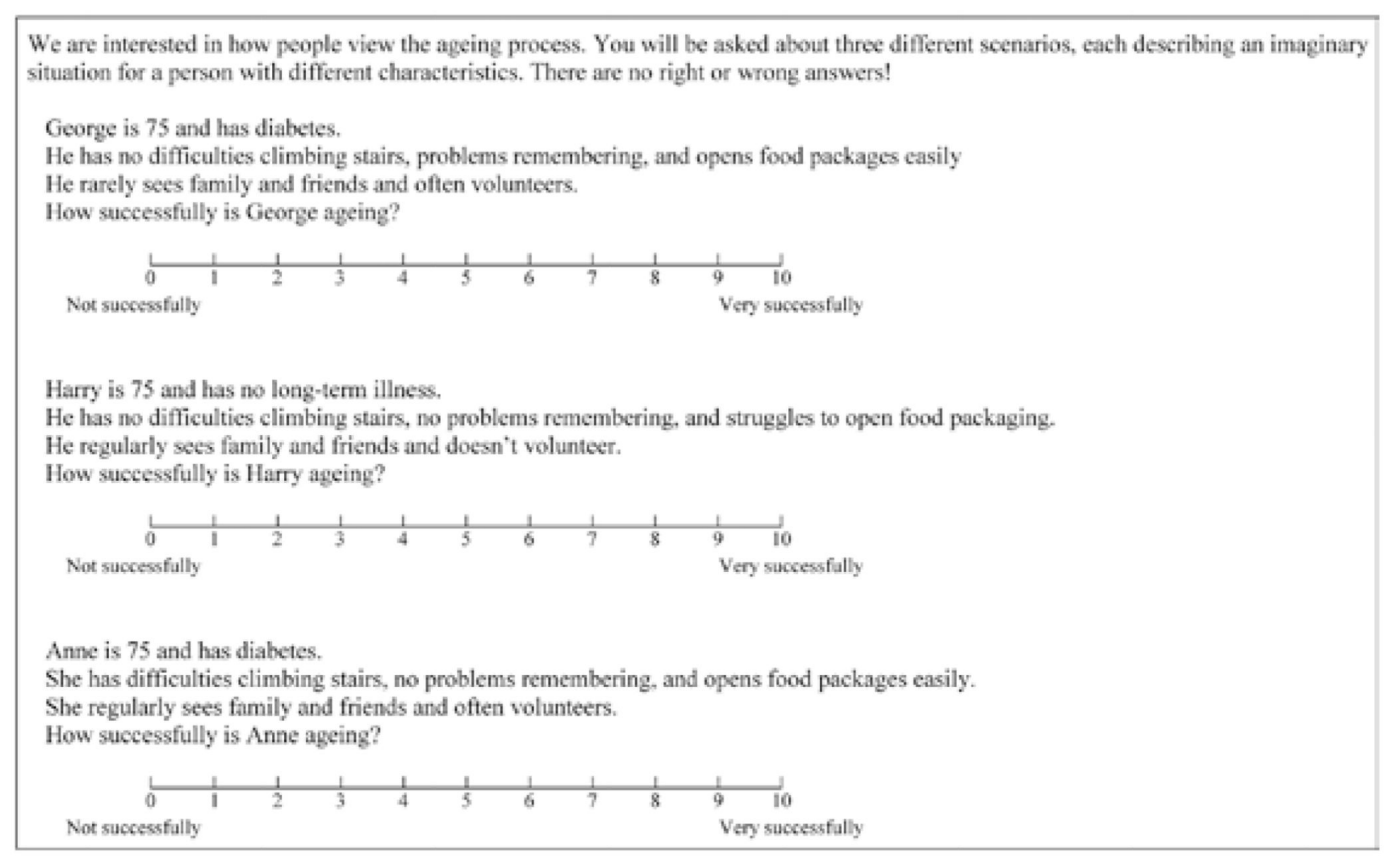
Introductory text and example vignettes as presented to respondents.

Vignettes were based on the six successful aging dimensions each with two possible outcomes (favorable vs unfavorable), resulting in a total of 2^6^ = 64 possible vignettes. Each respondent was presented with a set of three vignettes to allow comparison while avoiding the task becoming tedious or arduous. A 2^6^ factorial design was used to randomly (without replacement) generate these vignettes, ensuring that all combinations of favorable/unfavorable dimensions were equally represented across all respondent characteristics (Auspurg & Hinz, 2015). In addition, the randomization was designed to ensure that each respondent was presented with at least one male and one female vignette. Each vignette described a 75-year old with favorable/unfavorable outcomes for each of the six dimensions. The vignettes aimed to use neutral language, e.g., linking word “and” rather than “but,” to avoid directing responses. Definitions of favorable and unfavorable dimensions are presented in Table 1. These definitions were based on specific rather than general conditions and limitations to maintain realism and engagement with the exercise, e.g., focusing on “has difficulties climbing stairs,” rather than the broader “has a disability.” In addition, they were chosen to be easily recognized, understood, and realistic in the context of aging, e.g., considering productive engagement in terms of volunteering rather than paid employment. Finally, definitions aimed to be similar in terms of severity and open to interpretation in terms of their potential impact on successful aging. For example, diabetes was chosen as the chronic disease of interest as it is a leading cause of morbidity but can be successfully managed, whereas cancer might be regarded as more likely to be terminal and therefore more severe. After each vignette, respondents were asked “How successfully is [Name] aging?,” giving a score from 0 (not successfully) to 10 (very successfully). An example set of vignettes is shown in Figure 1 along with the introductory text presented to respondents.

Data from the experiment were analyzed using standard methods (Auspurg & Hinz, 2015). The relative importance of each vignette dimension in determining the successful aging score was assessed by comparing scores for all vignettes in which the dimension was favorable with scores for all vignettes in which it was unfavorable, regardless of the values of the other dimensions. Although, in the context of successful randomization, a simple comparison of means can be used, it is more usual (Auspurg & Hinz, 2015) to employ a multivariable (least squares) regression model in which all vignette dimensions are included simultaneously as independent binary (favorable versus unfavorable) predictors of the successful aging score. Moreover, when, as here, respondents are presented with multiple vignettes, random effects models are used to account for the hierarchical nature of the data (vignettes clustered within respondents) and the order in which vignettes are presented. In addition, in view of the survey design, the current analyses were also adjusted for sample and data collection mode and robust standard errors were calculated to allow for clustering within households and postcode sectors. As demonstrated in Supplementary Table 2, results from these regression models were very similar to those based on a simple comparison of means. Coefficients from the regression models for each dimension of interest measure the difference between the mean successful aging score across vignettes in which the dimension was favorable and the mean score across vignettes in which the dimension was unfavorable, with appropriate adjustments for the other dimensions and the study design. For example, the coefficient for (absence of) disease represents the (adjusted) difference between the mean score of all vignettes in which the individual was described as having no long-term illness and the mean score of all vignettes in which the individual was described as having diabetes. As each successful aging dimension was presented in the same way (favorable versus unfavorable), it is valid to make direct comparisons between them (Auspurg & Hinz, 2015) and the outcome measures from the models therefore represent the relative importance of each favorable dimension in determining the successful aging score. Formal comparisons of the relative importance of different dimensions were made post-estimation by considering linear combinations of regression coefficients (e.g., β_disease_ – β_disability_).

Analyses were repeated stratified by respondent gender, age group, long-standing illness, marital status, employment status, financial difficulties, satisfaction with health, satisfaction with income, satisfaction with leisure time, satisfaction with life, and by vignette gender to explore what impact these factors had on the relative importance attributed to each dimension. Formal statistical tests of effect modification by these factors were carried out by including appropriate interaction terms in the regression models. The six age groups included five younger than the person described in the vignette (<35, 35–44, 45–54, 55–64, 65–74), representing those anticipating the scenario with varying proximity, and one the same age or older (75+), considering the scenario concurrently or in retrospect.

In sensitivity analyses, all analyses were repeated using a subgroup of respondents for whom inverse probability weights were available. These weights are calculated by the Understanding Society Team to adjust for differential non-response, unequal selection probabilities, and differential sampling error so that findings from the Innovation Panel can be generalizable to the British population (Jäckle et al., 2017). Analyses using these weights were very similar to those presented here. An outline of the design and analysis was prepared and approved before data collection and is held by the Understanding Society Team.

## Results

**Figure 2:**
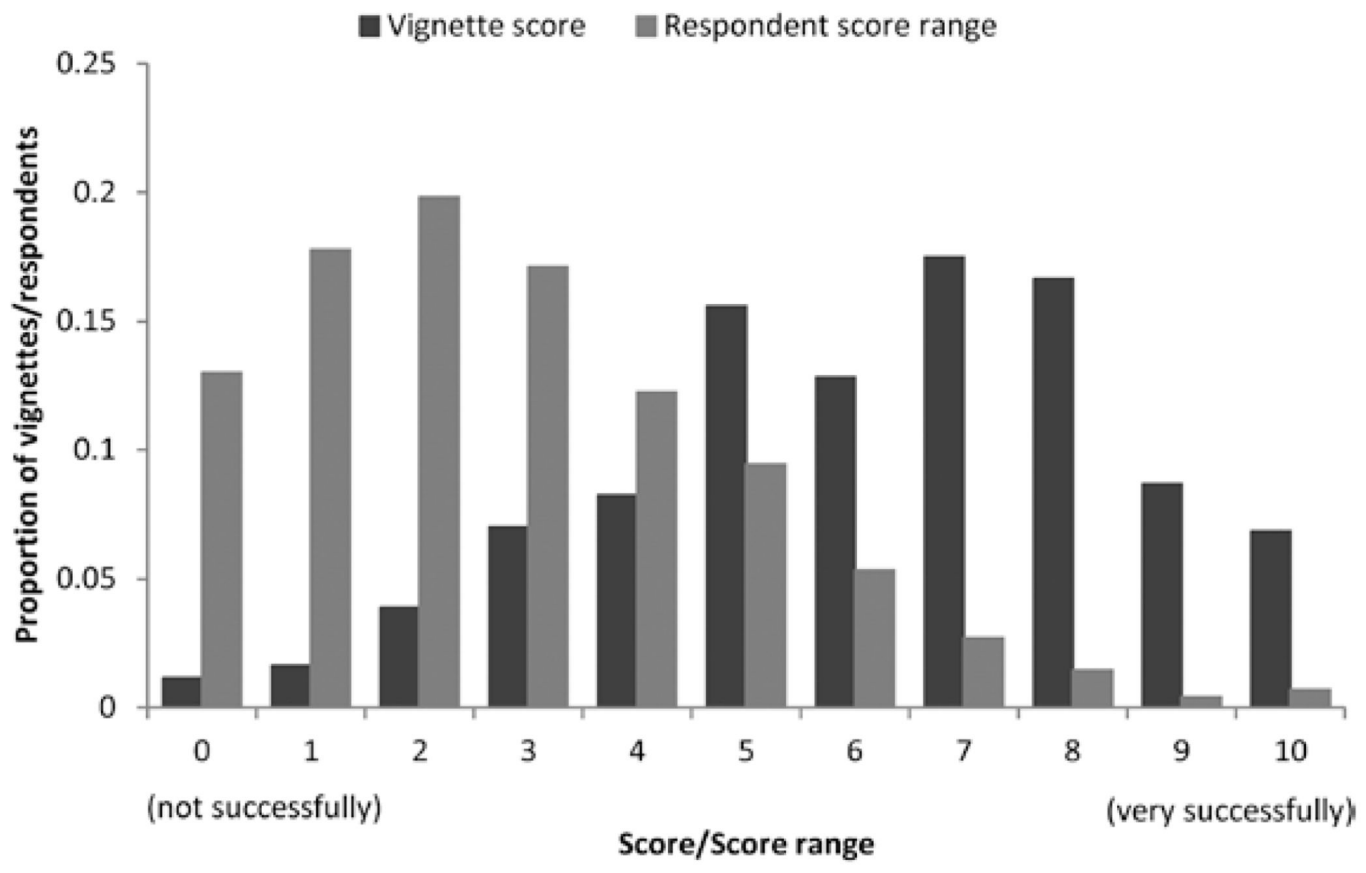
Vignette scores given in response to question “How successfully is [Name] aging?” (*N* = 5,967) and range of scores given by each respondent (*N* = 2,010).

A total of 1,508 eligible households were invited to participate in IP9 and 1,277 (85%) did so (Supplementary Figure 1). Within participating households, there were 2,545 eligible adults, 2,143 (84%) of whom took part in either web (*N* = 1,123) or face-to-face (*N* = 1,020) interviews; an additional 31 respondents had telephone interviews. Of those interviewed via the web or face-to-face, 2,010 (94%) took part (unaided) in the self-complete section, which contained the vignettes. The ages of those who took part ranged from 16 to 93 years. Characteristics of the respondents who were presented with the vignettes are presented in Supplementary Table 1. In total, 1,986 (99%) gave a score to all three and 24 (1%) to two or fewer. All scored vignettes were included in the analyses, giving a total of 5,967 completed overall. As would be expected from the design of the experiment, approximately half of all dimensions were favorable and the total of number of favorable dimensions in each vignette varied from none to six in approximately equal proportions. The success of the randomization is demonstrated by the similarities in the percentage of positive dimensions across all respondent characteristics. In addition, favorable dimensions were approximately equally allocated across vignettes describing men and women. The scores given to the vignettes are summarized in Figure 2 along with the range of scores given by each respondent across the three vignettes (i.e., the difference between the highest and lowest scoring vignette presented to the individual respondent). Individual vignette scores ranged from 0 to 10 and were somewhat skewed toward the upper (more successful) end of the range with a mean (standard deviation [*SD*]) score across all vignettes of 6.2 (2.3). The range of scores given by each respondent across the three vignettes also varied from 0 to 10, with a mean (*SD*) range of 2.8 (2.1). The good spread of vignette scores and respondent ranges indicate that respondents distinguished between the vignettes and did not simply allocate an average score to them all. In general, there was little evidence of systematic differences in mean scores according respondent characteristics and vignette gender (Supplementary Table 1) although there was some evidence to suggest that, overall, women allocated somewhat higher scores than men (mean [*SD*] score: 6.4 (2.3) vs 6.0 [2.3]) and that scores decreased slightly with respondent age (e.g., 6.3 [2.2] vs 5.9 [2.4] in <35 vs 75+ year olds, respectively).

**Figure 3:**
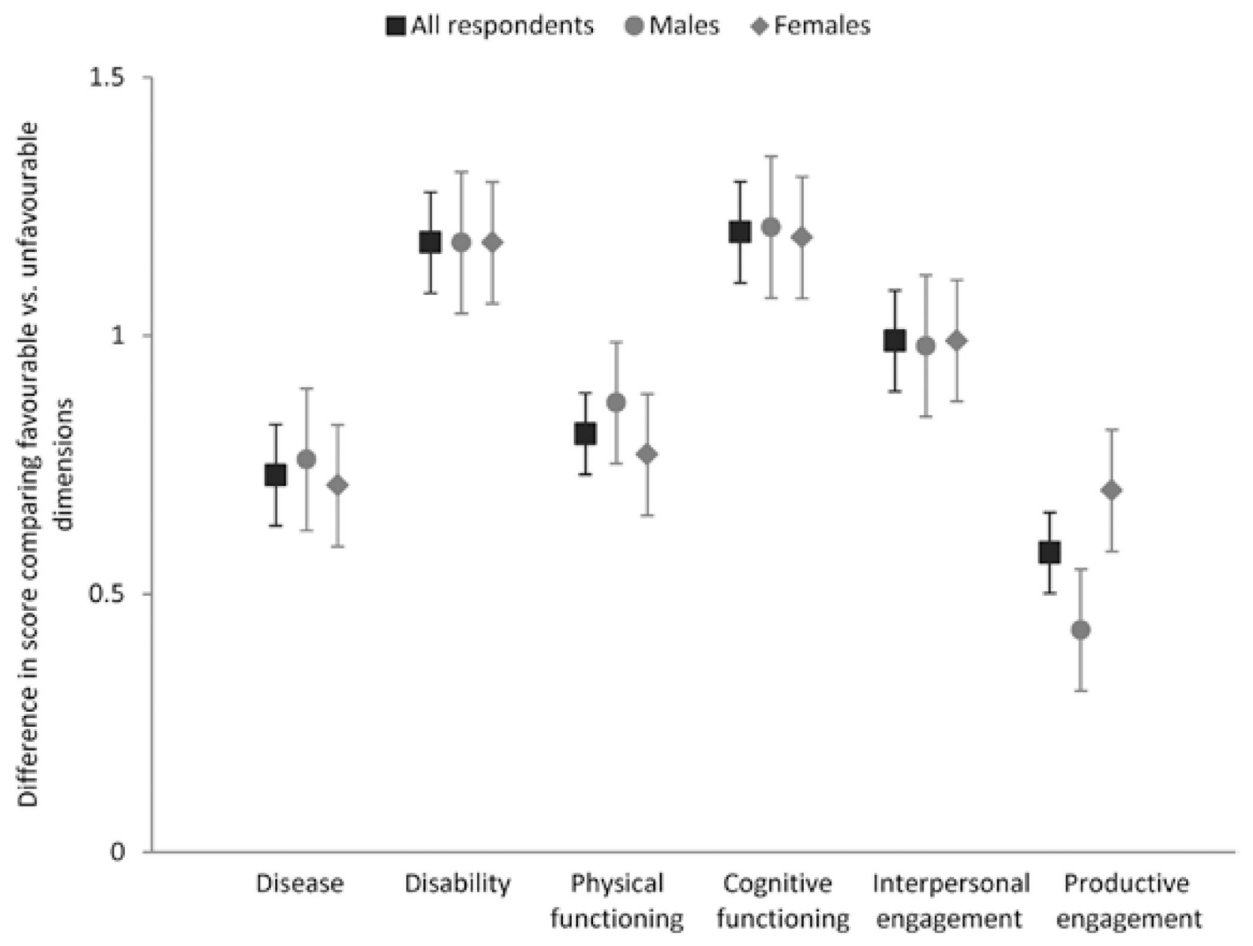
Relative importance of dimensions in determining successful aging score (based on difference (95% confidence interval) in mean successful aging score from regression model comparing vignettes with favorable versus unfavorable dimensions) for all respondents combined plus, separately, male and female respondents.

The importance given to each of the successful aging dimensions, based on coefficients from regression models, is presented in Figure 3. Numbers giving rise to this figure are presented in Supplementary Table 2 along with standardized effect sizes. Vignettes in which a particular dimension was favorable were consistently allocated higher scores than those in which the same dimension was unfavorable, with confidence intervals for the difference in mean scores excluding 0 (representing no impact of the dimension on successful aging scores) in every case. However, the relative importance of the dimensions varied. Differences in the weights given to the different successful aging dimensions are presented in Supplementary Table 3. The dimensions given the greatest importance by respondents were cognitive function and disability; vignettes in which these dimensions were favorable were allocated successful aging scores that were 1.20 (95% confidence interval [CI]: 1.11, 1.30) and 1.18 (1.08, 1.27) points respectively higher than those in which the dimensions were unfavorable, with identical corresponding standardized effect sizes of 0.56 (0.51, 0.61). Interpersonal engagement was also given relatively high importance (difference in mean scores: 0.99 [0.89, 1.08]; standardized effect size: 0.47 [0.42, 0.52]), although lower than disability and cognitive function (*p* for difference with cognitive function < .001). Disease and physical function were given similar importance overall (difference in mean scores: 0.73 [0.64, 0.82] and 0.81 [0.73, 0.90]; standardized effect size: 0.32 [0.27, 0.37] and 0.37 [0.32, 0.42], respectively) and, again, this was markedly lower than disability, cognitive function and interpersonal engagement (e.g., *p* for difference between disease and disability < .001). The dimension given least weight was productive engagement (difference in mean scores: 0.58 [0.49, 0.66]; standardized effect size: 0.27 [0.22, 0.32], *p* for difference with other dimensions < .02).

Responses to vignettes were consistent across vignette gender, and the majority of respondent characteristics (Supplementary Table 4). However, there was some evidence that women gave more importance to productive engagement than men (difference in mean scores for women and men: 0.70 [0.58, 0.81] vs 0.43 [0.30, 0.55], respectively; *p* for interaction with gender = .002), although productive engagement remained the dimension given least importance by both genders. In addition, there was a suggestion that respondents who were married or living with a partner gave somewhat less importance to (absence of) disease than those living alone (difference in mean scores: 0.64 [0.52, 0.76] vs 0.85 [0.72, 0.99], respectively; *p* = .02). Responses also differed somewhat between respondents who were retired versus those who were employed/unemployed but these differences were due to variation in responses by age and results for those who were employed and unemployed were very similar.

**Figure 4:**
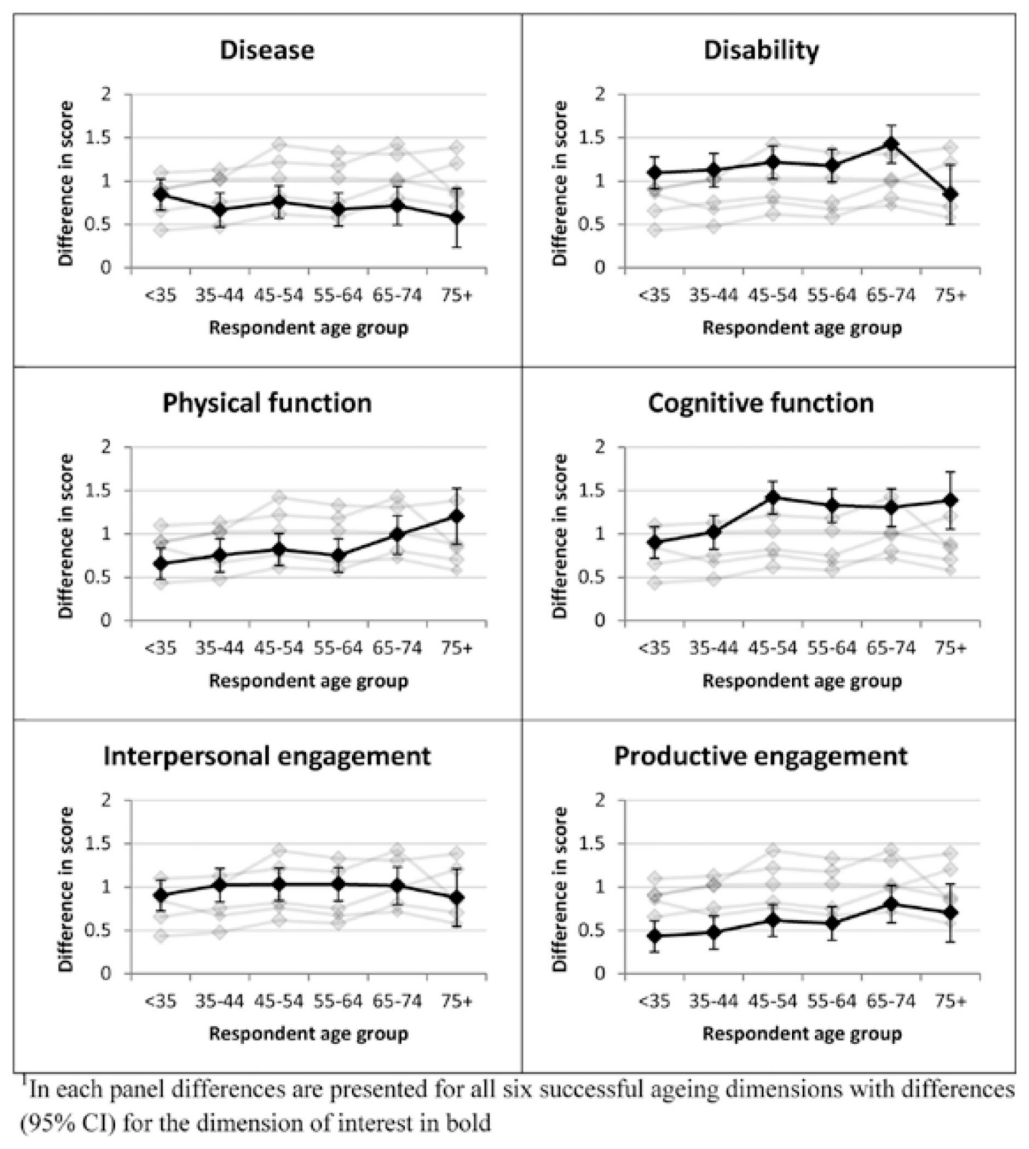
Relative importance of dimensions in determining successful aging score (based on difference in mean successful aging score from regression model comparing vignettes with favorable versus unfavorable dimensions) by age group. In each panel, differences are presented for all six successful aging dimensionswith differences (95% CI) for the dimension of interest in bold.

There were marked variations in the relative importance attributed to different dimensions by respondents of different ages, particularly for physical and cognitive function and productive engagement, as shown in Figure 4. In each panel, results are presented for all six successful aging dimensions, with differences (95% CI) in mean successful aging scores between favorable and unfavorable vignettes for the dimension of interest in bold. Numbers giving rise to this figure are presented in Supplementary Table 4. As previously observed in Figure 3, relative to other dimensions, disease was generally given low importance and this fell slightly, but not markedly, with increasing age so that mean differences between favorable and unfavorable vignettes in the oldest age groups (65–74 and 75+) were the smallest overall (difference in mean scores: 0.71 [0.47, 0.95] and 0.58 [0.23, 0.93], respectively; *p* for interaction with age group = .23). In contrast, disability was one of the dimensions given the greatest importance at almost all ages, and the most important among 65–74-year olds (difference in mean scores: 1.42 [1.19, 1.66]), although it was given somewhat less weight in 75+ year olds (difference in mean scores: 0.85 [0.49, 1.22]; *p* for interaction with age group = .39). Physical function was given relatively low weight by younger age groups (e.g., difference in mean scores among <35-year olds: 0.66 [0.47, 0.84]) but this increased with age, rising to one of the most important dimensions in 75+ year olds (difference in mean scores: 1.20 [0.88, 1.52]; *p* for interaction with age group = .003). Cognitive function was consistently given high importance relative to other dimensions, particularly in those aged 45+, and in 45–54, 55–64, and 75+ year olds was the most important overall (e.g., difference in mean scores in 75+ year olds: 1.39 [1.02, 1.76]; *p* for interaction with age group < .001). Interpersonal engagement was consistently in the middle of the dimensions in terms of importance (e.g., difference in mean scores in 45–54-year olds: 1.03 [0.81, 1.25]) and there was little evidence that this varied with respondent age (*p* for interaction with age group = .61). Finally, although productive engagement increased slightly in importance with age, overall it was given less weight than the other dimensions and, in respondents aged less than 65 years, differences in mean scores comparing favorable and unfavorable productive engagement were the smallest overall (e.g., difference in means scores in less than 35-year olds: 0.43 [0.25, 0.62]; *p* for interaction with age group = .01).

## Discussion

Successful aging scores given to the vignettes covered the full range of possibilities and there was variation in the scores allocated across the three vignettes presented to each respondent, indicating that respondents distinguished between the different scenarios. Scores were consistently higher for vignettes describing dimensions in favorable rather than unfavorable terms although the relative importance of each dimension varied. Disease (presence/absence of diabetes) was one of the dimensions given least weight in this experiment and the weight decreased with increasing age so that, among respondents aged 65+, disease was regarded as the least important overall. Productive engagement (volunteering) was also consistently less important than other dimensions, particularly among men, although scores increased at older ages. In contrast, disability (difficulties climbing stairs) and cognitive function (problems remembering) were given the greatest importance at all ages, with the exception of a drop in the disability weight among those aged 75+. Physical function (difficulties opening food packaging) was given relatively low weight by younger respondents but increased in importance in those aged 65+. Interpersonal engagement (meeting family and friends regularly) was consistently weighted in the middle. The relative importance given to the different dimensions were generally consistent across respondent characteristics other than age, and the gender of the vignette had no impact on the results.

Existing work considering societal attitudes toward successful aging has largely focused on qualitative responses to open-ended questions (Bowling, 2007; Cosco et al., 2013). The use of vignettes, in which respondents consider a fictitious third party, encourages individuals to consider successful aging as a broad hypothetical concept rather than asking whether they themselves are aging successfully. In addition, although respondents’ circumstances may influence their responses to vignettes the randomization of dimensions across vignettes ensures a balanced design, meaning that potential biases and confounding arising from differences in individual circumstances are eliminated. However, the experiment also has some limitations. The Innovation Panel is a household survey and individuals living in institutions are not included, although if individuals from previous waves move into an institution attempts are still made to interview them where appropriate. However, it is of note that results from analyses weighted to be representative of the British population were very similar to those presented here. The wide age range of respondents is a major strength of the experiment. However, in spite of the large sample size, it was necessary to base age-stratified analyses on six age groups, the youngest including 16–34 years and the oldest 75–93-year olds. These two age groups span almost 20 years each and there may be age-related differences within them that are not captured in these analyses. Future work might focus on narrower age bands but this would require substantially larger numbers of participants. It is also possible that the relative importance given to different dimensions was influenced by the success with which the definitions captured them. Definitions were based on common factors from existing, validated scales and were chosen to be easily recognized, understandable, and relevant to older individuals. In addition, the perceived severity of the definitions may have impacted on the results; for example a more life-limiting disease e.g., cancer, or a more severe disability, e.g., being in a wheelchair, might have been given greater importance than those described here. However, vignette definitions were selected to be similar in terms of their (limited) impact on activities of daily living. It is also worth noting that the relative importance given to the different dimensions in the present study are broadly consistent with existing literature. For example, a recent review of qualitative studies highlights the greater emphasis placed on psychosocial factors compared with physical health by older people (Cosco et al., 2013). Finally, many successful aging definitions, including the Rowe-Kahn model, have been criticized for not going far enough in capturing the priorities of older people, for example, well-being and autonomy (Ferri et al., 2009; Martinson & Berridge, 2015; Montross et al., 2006; Young et al., 2009). Although it would have been possible to include other dimensions such as these in our vignettes, this would have substantially increased the number required. In addition, our aim in this experiment was to specifically understand societal attitudes to the most commonly employed existing model of successful aging rather than create a new one that incorporates additional dimensions.

The value of patient preferences in directing clinical practice is well established but practitioners’ views may differ from those of their patients and this may be a particular problem in the context of aging as older patients are often reluctant to share their views (Joseph-Williams et al., 2017). The majority of vignettes in the current study described an individual with at least one unfavorable dimension who, according to standard definitions, would be considered *not* to be aging successfully. However, the mean score across all vignettes was well above the midpoint of the scale (toward “aging successfully”), suggesting that the general population have a positive view of aging, even in the context of disease, disability, or limitations of functioning and social engagement. The importance given to different successful aging dimensions was largely independent of respondents’ circumstances, e.g., there was no difference in the weights given to disease and disability among those with and without LSI and, similarly, no difference in the importance given to productive engagement in those who were employed or not. A specific criticism of the Rowe-Kahn model is that it reinforces social inequalities by defining successful aging as a state more easily achieved by those with higher socioeconomic position (Katz & Calasanti, 2015; Martinson & Berridge, 2015; Stowe & Cooney, 2015). However, our stratified analyses suggest that attitudes toward successful aging are not socially patterned, with almost identical results for those with and without financial difficulties and those who were satisfied or dissatisfied with their income. In addition, despite different experiences throughout the life course, results were largely consistent across vignette and respondents’ gender, although women gave somewhat more weight to volunteering, consistent with previous sociological work (Blaxter, 1990), perhaps reflecting traditional gender roles in this regard. In terms of individual successful aging dimensions, the consistently low importance given to disease reinforces qualitative findings in older individuals (Cosco et al., 2013), and extends these to younger ages. An isolated, possibly chance, finding from the current analysis suggests that respondents living alone gave more weight to disease than those in relationships, possibly reflecting greater perceived vulnerability in this group, although disease remained among the dimensions regarded as least important. In contrast, other biomedical dimensions, such as disability and cognitive function, along with interpersonal social engagement, were given some of the highest weights by respondents of all ages. Morbidity in older age is regarded as an important factor in determining health, social and economic policies, but policy makers and clinicians should recognize the relatively low value placed on disease by the general population and acknowledge the greater importance to individuals of good functioning and social engagement.

Perhaps the most striking results presented here are those demonstrating how attitudes to successful aging vary with age. The majority of existing work on perceptions of successful aging has focused on older people, while many researchers, clinicians and policy makers are younger than those under study. Understanding the views of younger individuals and how these differ from those at older ages has the potential to close the gap between the attitudes of clinicians and their patients and to promote shared decision making. For example, both physical function and productive engagement were viewed as relatively unimportant by those of working age (<65 years) but their weights increased among older respondents, consistent with previous work on lay perceptions of health (Blaxter, 1990). This highlights the potential for relatively common problems of older age, such as struggling with food packaging or lacking a meaningful role in society, to be dismissed by those involved in the care of older people. In contrast, the importance given to interpersonal engagement was very similar across all age groups, underlining the ubiquity of this dimension throughout the lifecourse and highlighting the need for health and social care services to, not only treat disease and poor functioning, but also create opportunities for social interaction. Moreover, it is important to recognize that attitudes among older people may continue to change as they age and that patient-centered care is an evolving process. For example, there was a sharp drop in the importance given to disability in the 75+ group, following steady rises at younger ages. This is an isolated finding and could be due to chance but could also reflect shifting attitudes toward disability in an age group who were “living the vignette” and, perhaps, beginning to experience, and therefore recognize, physical decline. Clinicians wishing to base their practice on shared decision making and patient-centered care should recognize potential differences between their own priorities for successful aging and those of their patients and, while there is no substitute for face-to-face discussion with patients, our results provide guidelines as to how these may differ.

Results from this study support and extend existing work, providing unconfounded estimates of the relative importance given to six successful aging dimensions by a large U.K. population sample and demonstrating how these vary across the lifecourse. However, it is not clear whether wider societal policies such as health and social care or pension provision influence these results and it would be of considerable interest to repeat this experiment in other populations where these differ. In addition, given changing attitudes with age, it would be beneficial to understand how major life events such as retirement or bereavement influence these results. As well as informing clinicians and policy makers working directly with older people, our results are relevant to researchers interested in measuring successful aging and its determinants. The Rowe-Kahn definition of successful aging is a widely used research tool with “success” traditionally defined as a dichotomy in which all six criteria are met although, in practice, very few older people achieve this, despite considering themselves to be aging well (McLaughlin et al., 2012; Montross et al., 2006; Strawbridge et al., 2002). A more pragmatic approach has been proposed in which the *extent* of success in aging is measured by summing the number of favorable dimensions (Bowling, 2007; Bowling & Iliffe, 2011; Whitley et al., 2016), and our results may provide a more “cutting edge” approach (Gu et al., 2017) in which favorable dimensions are weighted according to the priorities of the general population. Such a measure would provide a more nuanced approach to successful aging and acknowledges the importance of quality as well as quantity of life, consistent with challenges to the notion of health as complete physical, mental and social well-being (Huber et al., 2011). The quality-adjusted life year (QALY), which weights different health states according to patient preference, is a well-established health outcome (Whitehead & Ali, 2010). A similar measure based on weights such as those presented here might form the basis for a modified Rowe-Kahn model (Rowe & Kahn, 2015) that better represents societal attitudes toward successful aging and could be used to evaluate interventions and direct policy investments to promote successful aging worldwide.

## Supplementary Material

Supplementary data is available at *The Journals of Gerontology, Series B: Psychological Sciences and Social Sciences* online.

Supplement

## Figures and Tables

**Table 1:** Favorable and Unfavorable Rowe-Kahn Successful Aging Dimensions Used in the Vignettes

Successful aging dimension	Favorable	Unfavorable	Details
Disease	No long-term illness	Diabetes	Diabetes is a common disease of old age that is well known, doesn’t typically affect physical functioning, and avoids the potential life-limiting connotations of, for example, cancer or heart disease.
Disability	No difficulties climbing stairs	Difficulties climbing stairs	Difficulties with stairs is included in many health and disability scales, e.g., SF-36, Lambeth Diasability Screening Questionnaire, OECD Long-term Disability Questionnaire (McDowell, 2006).
Physical functioning	Opens food packages easily	Struggles to open food packaging	Problems with opening food packaging is included in several functional status scales, e.g., Functional Status Index, Stanford Health Assessment Questionnaire (McDowell, 2006).
Cognitive functioning	No problems remembering	Problems remembering	Memory forms an integral part of many cognitive tests and, in the context of aging, loss of memory is a prominent feature of dementia.
Interpersonal engagement	Regularly sees friends and family	Rarely sees friends and family	Frequency of contact with family and friends is commonly used in social health scales, e.g., RAND Social Health Battery, Katz Adjustment Scale (McDowell, 2006).
Productive engagement	Often volunteers	Doesn’t volunteer	Volunteering is a common form of productive engagement in the age group covered by the vignette, who are generally past retirement age.
