# Supplementary material for: Population Priorities for Successful Aging: A Randomized Vignette Experiment
Source: J Gerontol B Psychol Sci Soc Sci. Author manuscript; Available in PMC 2020 Jan 21. (PMC6974399; doi:10.1093/geronb/gby060)
Supplement: Supplement [file EMS84155-supplement-Supplement.docx]

Supplementary Figure 1: Construction of analytical sample (N^H^, N^P^, and N^V^ refer to number of households, persons, and vignettes respectively)

|  | | |  | | | |  | Household sample  N^H^=1,508 | | | | | | | | | | | | |  | |  | | | | |  |  | | |
| --- | --- | --- | --- | --- | --- | --- | --- | --- | --- | --- | --- | --- | --- | --- | --- | --- | --- | --- | --- | --- | --- | --- | --- | --- | --- | --- | --- | --- | --- | --- | --- |
|  |  |  |  |  |  |  |  |  |  |  |  |  |  |  |  |  |  |  |  |  |  |  |  |  |  |  |  |  |  | | |
|  | | |  | | | |  |  | | | | |  | | | | | | | |  | |  | | | | |  | *No contact: 95* | | |
|  |  |  |  |  |  |  |  |  | | | | |  | | | | | | | |  |  |  |  |  |  |  |  | *Ineligible: 59* | | |
|  | | |  | | | |  |  | | | | |  | | | | | | | |  | |  | | | | |  | *Refusal: 77* | | |
|  |  |  |  |  |  |  |  |  |  |  |  |  |  |  |  |  |  |  |  |  |  |  |  |  |  |  |  |  |  | | |
|  | | |  | | | |  | Participating households  N^H^=1,277 | | | | | | | | | | | | |  | |  | | | | |  |  | | |
|  |  |  |  |  |  |  |  |  |  |  |  |  |  |  |  |  |  |  |  |  |  |  |  |  |  |  |  |  |  | | |
|  | | |  | | | |  | | | |  | | |  | |  | | | | | | |  | | | | |  | | |  |
|  | | |  | | | |  | Eligible adults  N^P^=2,545 | | | | | | | | | | | | |  | |  | | | | |  |  | | |
|  |  |  |  |  |  |  |  |  |  |  |  |  |  |  |  |  |  |  |  |  |  |  |  |  |  |  |  |  | *No response/interview: 174*  *Ineligible: 15*  *Refusal: 119* | | |
|  | | |  | | | |  | | | |  | | |  | | |  | | | | |  | | |  | | | |  |  |  |
|  |  |  |  |  |  |  |  |  |  |  |  |  |  |  | | |  | | | | |  | | |  | | | | *Proxy interview: 63*  *Telephone interview: 31* | | |
|  | | |  | |  | | | Participating adults  N^P^=2,143 | | | | | | | | | | | | |  | | | |  | | |  |  | | |
|  |  |  |  | |  | | |  |  |  |  |  |  |  |  |  |  |  |  |  |  | | | |  | | |  |  | | |
|  | | |  | |  | | | | | |  | | | | |  | | | | | | | | |  | | |  |  | | |
|  | Web interview  N^P^=1,123 | | | | | | | | |  |  | | | | |  | | Face to face interview  N^P^=1,020 | | | | | | | | | | |  | |  |
|  | | |  | |  | |  | |  | | | | | | | | | | | |  | |  | |  | |  | |  | | |
|  |  |  |  |  |  | |  | | *Exited survey before SC: 5* | | | | | | | | | | | |  |  |  |  |  | |  | | *Unable to do SC unaided:115*  *Refusal: 13* | | |
|  |  |  |  |  |  | |  | |  |  |  |  |  |  |  |  |  |  |  |  |  |  |  |  |  | |  | |  |  |  |
|  |  |  |  |  |  | |  | |  | | | | | | | | | | | |  |  |  |  |  | |  | |  | | |
|  | Self-completion accepted  N^P^=1,118 | | | | | | | | |  |  | | | | |  | | Self-completion accepted  N^P^=892 | | | | | | | | | | |  | |  |
|  | | | | |  | | | |  | |  | | | | |  | | | |  | | | | |  | | | | | |  |
|  | | |  | |  |  | | Presented with vignettes  N^P^=2,010 | | | | | | | | | | | | |  | | |  |  | | |  | | |  |
|  |  |  |  | |  |  | |  |  |  |  |  |  |  |  |  |  |  |  |  |  | | |  |  |  |  |  |  |  |  |
|  | | |  | | | |  | | | |  | | |  | |  | | | | | | |  | | | | |  | | |  |
|  | |  | | | | | | | |  | | | | | | | | |  | | | | | | | | | |  | |  |
| No vignettes scored  N^P^=18 | | | |  | | | One vignette scored  N^P^=3 | | | | |  | | | Two vignettes scored  N^P^=3 | | | | | | | | |  | | Three vignettes scored  N^P^=1,986 | | | |  | |
|  | |  | | | | | | | |  | | | | | | | | |  | | | | | | | | | |  | |  |
|  | | |  | | | |  | | | |  | | |  | |  | | | | | | |  | | | | |  | | |  |
|  | | |  | | | |  | Completed vignettes  N^V^=5,967 | | | | | | | | | | | | |  | |  | | | | |  | | |  |

Supplementary Table 1: Characteristics of respondents and vignettes

|  |  |  | % of vignettes with favourable dimension | | | | | |  |
| --- | --- | --- | --- | --- | --- | --- | --- | --- | --- |
|  | N respondents | N vignettes | Disease | Disability | Physical function | Cognitive function | Interpersonal engagement | Productive engagement | Mean (SD) score |
|  |  |  |  |  |  |  |  |  |  |
| **Respondent characteristics** |  |  |  |  |  |  |  |  |  |
| Male  Female | 906  1,104 | 2,690  3,277 | 50.6  51.5 | 51.6  50.0 | 50.5  50.0 | 49.6  51.7 | 49.2  50.1 | 49.5  50.9 | 6.0 (2.3)  6.4 (2.3) |
|  |  |  |  |  |  |  |  |  |  |
| <35  35-44  45-54  55-64  65-74  75+ | 459  316  401  389  306  139 | 1,357  936  1,187  1,161  911  415 | 51.1  48.3  52.7  51.9  51.8  49.4 | 50.0  51.1  49.7  51.4  51.6  51.1 | 48.3  50.8  51.5  51.2  50.0  49.6 | 51.9  49.9  50.6  51.3  51.6  45.5 | 48.6  49.0  50.6  49.4  51.3  49.6 | 50.1  49.4  52.5  47.0  52.7  50.6 | 6.3 (2.2)  6.3 (2.2)  6.1 (2.3)  6.2 (2.4)  6.2 (2.3)  5.9 (2.4) |
|  |  |  |  |  |  |  |  |  |  |
| No LSI  LSI^*^ | 1,301  707 | 3,860  2,101 | 50.4  52.4 | 50.8  50.7 | 49.8  51.0 | 50.9  50.3 | 49.9  49.4 | 50.4  50.3 | 6.2 (2.3)  6.1 (2.3) |
|  |  |  |  |  |  |  |  |  |  |
| Married or partner  No partner^*^ | 1,117  881 | 2,615  3,320 | 52.0  50.5 | 51.3  50.3 | 49.7  50.8 | 51.2  50.5 | 48.8  50.4 | 50.0  50.6 | 6.2 (2.3)  6.2 (2.3) |
|  |  |  |  |  |  |  |  |  |  |
| Employed  Not employed  Retired^*^ | 1,166  319  520 | 3,451  950  1,551 | 50.8  51.8  51.3 | 50.0  52.4  51.2 | 49.8  50.3  51.3 | 51.6  51.6  48.6 | 49.2  49.3  51.3 | 50.3  48.2  51.6 | 6.2 (2.2)  6.3 (2.3)  6.2 (2.4) |
|  |  |  |  |  |  |  |  |  |  |
| No financial difficulties  Financial difficulties^*^ | 1,856  144 | 5,516  432 | 51.1  50.5 | 50.9  48.8 | 50.2  50.5 | 50.8  50.0 | 49.7  49.8 | 50.2  51.2 | 6.2 (2.3)  6.2 (2.5) |
|  |  |  |  |  |  |  |  |  |  |
| Satisfied with health  Dissatisfied with health^*^ | 1,541  460 | 4,583  1,366 | 51.2  50.4 | 50.8  50.9 | 50.1  50.8 | 50.8  50.4 | 50.4  47.4 | 50.7  48.6 | 6.2 (2.3)  6.1 (2.3) |
|  |  |  |  |  |  |  |  |  |  |
| Satisfied with income  Dissatisfied with income^*^ | 1,542  455 | 4,587  1,358 | 51.0  51.2 | 50.8  50.6 | 50.3  50.3 | 51.0  49.9 | 50.1  48.5 | 50.3  50.4 | 6.3 (2.8)  6.2 (2.3) |
|  |  |  |  |  |  |  |  |  |  |
| Satisfied with leisure time  Dissatisfied with leisure time^*^ | 1,507  491 | 4,482  1,463 | 51.2  50.7 | 50.6  51.3 | 50.0  51.1 | 50.5  51.6 | 49.8  49.5 | 50.3  50.2 | 6.2 (2.3)  6.1 (2.3) |
|  |  |  |  |  |  |  |  |  |  |
| Satisfied with life  Dissatisfied with life^*^ | 1,769  232 | 5,260  689 | 51.1  50.7 | 51.0  49.1 | 50.0  52.0 | 50.7  51.2 | 49.8  48.9 | 50.5  48.5 | 6.2 (2.3)  6.0 (2.4) |
|  |  |  |  |  |  |  |  |  |  |
| **Vignette characteristics** |  |  |  |  |  |  |  |  |  |
| Male  Female | - | 2,966  3,001 | 51.5  50.8 | 50.4  51.0 | 50.5  50.0 | 50.3  51.1 | 49.3  50.1 | 51.2  49.5 | 6.2 (2.3)  6.2 (2.3) |
|  |  |  |  |  |  |  |  |  |  |

^*^Numbers do not match total due to missing values

Supplementary Table 2: Difference (95% CI) in mean successful ageing score comparing vignettes with favourable versus unfavourable dimensions using simple comparison of means and regression analyses

| Vignette dimension | Simple comparison of means | | Multivariable random effects regression model with robust standard errors plus adjustment for sample and interview mode | | Standardised effect size  (Cohen’s d) | |  |
| --- | --- | --- | --- | --- | --- | --- | --- |
|  |  | |  | |  | |  |
| Disease | 0.72 (0.61, 0.84) | | 0.73 (0.64, 0.82) | | 0.32 (0.27, 0.37) | |  |
| Disability | 1.23 (1.12, 1.35) | | 1.18 (1.08, 1.27) | | 0.56 (0.51, 0.61) | |  |
| Physical function | 0.83 (0.71, 0.94) | | 0.81 (0.73, 0.90) | | 0.37 (0.32, 0.42) | |  |
| Cognitive function | 1.24 (1.12, 1.35) | | 1.20 (1.11, 1.30) | | 0.56 (0.51, 0.61) | |  |
| Interpersonal engagement | 1.06 (0.94, 1.17) | | 0.99 (0.89, 1.08) | | 0.47 (0.42, 0.52) | |  |
| Productive engagement | 0.61 (0.50, 0.73) | | 0.58 (0.49, 0.66) | | 0.27 (0.22, 0.32) | |  |
|  |  |  | |  | |  |  |

Supplementary Table 3: Pairwise differences (95% confidence intervals) in relative importance given to successful ageing dimensions based on linear combinations of regression coefficients

|  | Disease | Disability | Physical function | Cognitive function | Interpersonal engagement |
| --- | --- | --- | --- | --- | --- |
|  |  |  |  |  |  |
| Disability | -0.44 (-0.57, -0.32) |  |  |  |  |
|  | <0.001 |  |  |  |  |
|  |  |  |  |  |  |
| Physical function | -0.08 (-0.21, 0.05) | 0.36 (0.24, 0.48) |  |  |  |
|  | 0.21 | <0.001 |  |  |  |
|  |  |  |  |  |  |
| Cognitive function | -0.47 (-0.59, -0.35) | -0.02 (-0.15, 0.10) | -0.39 (-0.51, -0.27) |  |  |
|  | <0.001 | 0.66 | <0.001 |  |  |
|  |  |  |  |  |  |
| Interpersonal engagement | -0.26 (-0.38, -0.13) | 0.19 (0.06, 0.31) | -0.17 (-0.30, -0.05) | 0.22 (0.08, 0.35) |  |
|  | <0.001 | 0.004 | 0.01 | 0.001 |  |
|  |  |  |  |  |  |
| Productive engagement | 0.15 (0.03, 0.28) | 0.60 (0.47, 0.72) | 0.24 (0.12, 0.35) | 0.63 (0.50, 0.75) | 0.41 (0.29, 0.53) |
|  | 0.02 | <0.001 | <0.001 | <0.001 | <0.001 |
|  |  |  |  |  |  |

Supplementary Table 4: Difference (95% CI) in mean successful ageing score from regression model comparing vignettes with favourable versus unfavourable dimensions stratified by respondent characteristics and vignette gender

|  | Difference (95% CI) in score | | | | | |
| --- | --- | --- | --- | --- | --- | --- |
|  | Disease | Disability | Physical function | Cognitive function | Interpersonal engagement | Productive engagement |
|  |  |  |  |  |  |  |
| **Respondent characteristics** |  |  |  |  |  |  |
| Male | 0.76 (0.63, 0.89) | 1.18 (1.04, 1.32) | 0.87 (0.74, 0.99) | 1.21 (1.07, 1.34) | 0.98 (0.85, 1.11) | 0.43 (0.30, 0.55) |
| Female | 0.71 (0.59, 0.83) | 1.18 (1.05, 1.30) | 0.77 (0.66, 0.89) | 1.19 (1.07, 1.32) | 0.99 (0.86, 1.12) | 0.70 (0.58, 0.81) |
| p^2^ | 0.54 | 0.98 | 0.31 | 0.89 | 0.94 | 0.002 |
|  |  |  |  |  |  |  |
| <35  35-44  45-54  55-64  65-74  75+ | 0.84 (0.65, 1.04)  0.67 (0.45, 0.88)  0.76 (0.56, 0.95)  0.67 (0.47, 0.88)  0.71 (0.47, 0.95)  0.58 (0.23, 0.93) | 1.09 (0.88, 1.29)  1.12 (0.92, 1.32)  1.22 (1.01, 1.42)  1.18 (0.97, 1.40)  1.42 (1.19, 1.66)  0.85 (0.49, 1.22) | 0.66 (0.47, 0.84)  0.75 (0.55, 0.95)  0.82 (0.65, 1.00)  0.75 (0.55, 0.96)  0.99 (0.77, 1.21)  1.20 (0.88, 1.52) | 0.90 (0.72, 1.08)  1.02 (0.80, 1.23)  1.42 (1.20, 1.63)  1.32 (1.11, 1.53)  1.30 (1.05, 1.55)  1.39 (1.02, 1.76) | 0.90 (0.72, 1.09)  1.02 (0.81, 1.23)  1.03 (0.81, 1.25)  1.03 (0.81, 1.25)  1.01 (0.78, 1.25)  0.88 (0.50, 1.25) | 0.43 (0.25, 0.62)  0.47 (0.29, 0.66)  0.61 (0.43, 0.80)  0.58 (0.37, 0.79)  0.80 (0.59, 1.02)  0.70 (0.37, 1.03) |
| p^2^ | 0.23 | 0.39 | 0.003 | <0.001 | 0.61 | 0.01 |
|  |  |  |  |  |  |  |
| Married or partner | 0.64 (0.52, 0.76) | 1.15 (1.03, 1.28) | 0.84 (0.73, 0.95) | 1.22 (1.10, 1.35) | 0.98 (0.85, 1.10) | 0.64 (0.53, 0.76) |
| No partner^*^ | 0.85 (0.72, 0.99) | 1.20 (1.06, 1.34) | 0.79 (0.66, 0.92) | 1.19 (1.05, 1.33) | 0.99 (0.85, 1.14) | 0.50 (0.37, 0.63) |
| p^2^ | 0.02 | 0.61 | 0.54 | 0.73 | 0.90 | 0.12 |
|  |  |  |  |  |  |  |
| Employed | 0.75 (0.63, 0.86) | 1.14 (1.02, 1.25) | 0.74 (0.63, 0.85) | 1.20 (1.08, 1.33) | 1.00 (0.87, 1.12) | 0.52 (0.41, 0.63) |
| Not employed | 0.77 (0.54, 0.99) | 1.11 (0.87, 1.36) | 0.57 (0.33, 0.81) | 0.94 (0.71, 1.18) | 0.94 (0.70, 1.17) | 0.55 (0.34, 0.77) |
| Retired^*^ | 0.68 (0.49, 0.87) | 1.29 (1.11, 1.48) | 1.08 (0.92, 1.25) | 1.35 (1.16, 1.54) | 1.00 (0.82, 1.18) | 0.75 (0.58, 0.92) |
| p^2^ | 0.61 | 0.18 | 0.002 | 0.35 | 1.00 | 0.03 |
|  |  |  |  |  |  |  |
| No LSI | 0.78 (0.67, 0.89) | 1.20 (1.09, 1.31) | 0.80 (0.70, 0.90) | 1.18 (1.07, 1.30) | 1.00 (0.88, 1.11) | 0.56 (0.46, 0.67) |
| LSI^*^ | 0.65 (0.50, 0.81) | 1.13 (0.98, 1.28) | 0.84 (0.69, 0.98) | 1.24 (1.08, 1.40) | 0.98 (0.82, 1.13) | 0.61 (0.47, 0.75 |
| p^2^ | 0.21 | 0.48 | 0.70 | 0.51 | 0.90 | 0.60 |
|  |  |  |  |  |  |  |
| No financial difficulties | 0.73 (0.63, 0.83) | 1.15 (1.04, 1.25) | 0.80 (0.71, 0.90) | 1.23 (1.12, 1.33) | 0.94 (0.84, 1.05) | 0.59 (0.49, 0.68) |
| Financial difficulties^*^ | 0.73 (0.55, 0.91) | 1.27 (1.08, 1.45) | 0.83 (0.66, 1.00) | 1.14 (0.96, 1.32) | 1.09 (0.92, 1.27) | 0.55 (0.38, 0.72) |
| p^2^ | 0.98 | 0.28 | 0.82 | 0.40 | 0.15 | 0.71 |
|  |  |  |  |  |  |  |
| Satisfied with health | 0.70 (0.58, 0.83) | 1.23 (1.11, 1.36) | 0.75 (0.63, 0.86) | 1.20 (1.06, 1.33) | 1.02 (0.89, 1.15) | 0.64 (0.53, 0.76) |
| Dissatisfied with health^*^ | 0.77 (0.64, 0.90) | 1.12 (0.99, 1.25) | 0.89 (0.77, 1.01) | 1.22 (1.09, 1.36) | 0.95 (0.82, 1.08) | 0.51 (0.39, 0.63) |
| p^2^ | 0.49 | 0.20 | 0.08 | 0.78 | 0.48 | 0.13 |
|  |  |  |  |  |  |  |
| Satisfied with income | 0.71 (0.58, 0.84) | 1.16 (1.02, 1.29) | 0.83 (0.70, 0.96) | 1.15 (1.01, 1.29) | 0.95 (0.82, 1.09) | 0.57 (0.44, 0.69) |
| Dissatisfied with income^*^ | 0.76 (0.63, 0.88) | 1.19 (1.07, 1.32) | 0.80 (0.69, 0.92) | 1.25 (1.12, 1.37) | 1.01 (0.89, 1.14) | 0.59 (0.48, 0.70) |
| p^2^ | 0.61 | 0.71 | 0.81 | 0.35 | 0.51 | 0.78 |
|  |  |  |  |  |  |  |
| Satisfied with leisure time | 0.65 (0.52, 0.79) | 1.20 (1.06, 1.34) | 0.87 (0.74, 1.00) | 1.25 (1.11, 1.39) | 1.06 (0.93, 1.20) | 0.63 (0.50, 0.76) |
| Dissatisfied with leisure time^*^ | 0.81 (0.68, 0.93) | 1.16 (1.04, 1.28) | 0.77 (0.66, 0.88) | 1.18 (1.06, 1.31) | 0.94 (0.81, 1.06) | 0.54 (0.42, 0.65) |
| p^2^ | 0.10 | 0.69 | 0.27 | 0.44 | 0.15 | 0.27 |
|  |  |  |  |  |  |  |
| Satisfied with life | 0.68 (0.57, 0.79) | 1.20 (1.07, 1.32) | 0.82 (0.71, 0.92) | 1.29 (1.17, 1.41) | 0.99 (0.87, 1.11) | 0.61 (0.50, 0.72) |
| Dissatisfied with life^*^ | 0.82 (0.67, 0.97) | 1.15 (1.00, 1.29) | 0.82 (0.69, 0.95) | 1.09 (0.94, 1.23) | 0.99 (0.84, 1.14) | 0.53 (0.39, 0.66) |
| p^2^ | 0.16 | 0.62 | 0.94 | 0.03 | 0.99 | 0.32 |
|  |  |  |  |  |  |  |
| **Vignette characteristics** |  |  |  |  |  |  |
| Male | 0.72 (0.59, 0.86) | 1.13 (1.00, 1.26) | 0.79 (0.66, 0.92) | 1.13 (1.00, 1.26) | 0.98 (0.84, 1.11) | 0.55 (0.42, 0.69) |
| Female | 0.80 (0.67, 0.93) | 1.21 (1.07, 1.34) | 0.80 (0.67, 0.93) | 1.27 (1.14, 1.41) | 1.05 (0.91, 1.18) | 0.62 (0.50, 0.75) |
| p^2^ | 0.56 | 0.38 | 0.83 | 0.42 | 0.50 | 0.62 |
|  |  |  |  |  |  |  |

^1^From multivariable random effects regression model with robust standard errors plus adjustment for sample and interview mode; ^2^p for interaction/effect modification by respondent characteristic and vignette gender
